# Association Between Vitamin D, Zinc, and Thyroid Biomarker Levels With Vitiligo Disease: A Retrospective Cohort Study in a Tertiary Care Center

**DOI:** 10.7759/cureus.31774

**Published:** 2022-11-22

**Authors:** Bader Bashrahil, Ziyad Alzahrani, Mohammad Nooh, Nawaf Alghamdi, Hatem Alsolami, Rahaf Alturkistani, Siham Marghalani

**Affiliations:** 1 College of Medicine, King Saud Bin Abdulaziz University for Health Sciences, Jeddah, SAU; 2 Department of Dermatology, Ministry of the National Guard-Health Affairs, Jeddah, SAU

**Keywords:** vit-d, nonsegmental, segmental, thyroid, vitiligo, zinc

## Abstract

Objectives

Vitiligo is a dermatological autoimmune disease that has been linked with numerous risk factors. There is an elevated level of evidence suggesting a linkage between vitiligo disease and zinc, vitamin D (Vit-D), thyroid hormones, and thyroid antibody levels.

Methods

This retrospective cohort study included patients of all age groups of both sexes. Patients were investigated for demographics, vitiligo characteristics, and laboratory tests, including zinc, Vit-D, T3 (triiodothyronine), T4 (thyroxine), thyroid-stimulating hormone (TSH), thyroid peroxidase antibody (TPOAb), and thyroglobulin antibody (TGAb).

Results

Two hundred and ninety-seven patients were retrospectively assessed; they averaged 29 years for segmental vitiligo (SV) and 31 years for nonsegmental vitiligo (NSV). Gender-wise, our study included more females (57.5%) than males (42.5%). Females comprised approximately 51.8% of NSV patients, while males constituted 36.7%. Patients' T3, T4, and TPOAb levels correlated significantly with age (p=0.001, p <0.01, p=0.14), and elevated BMI recorded high TPOAb levels (p<0.001). An increase in TGAb was associated with extensive involvement in the depigmentation of body surface area (BSA). The segmental type had the lowest TGAb and TPOAb titers. The universal subtype of vitiligo recorded the highest TSH, T3, and TGAb levels. However, differences in laboratory test levels were insignificant for the sex, the type of vitiligo, or the subtype of vitiligo.

Conclusion

In conclusion, neither Vit-D nor zinc had a significant linkage with any of vitiligo's characteristics or treatments. Nonetheless, TGAb had a significant correlation to the BSA involved with vitiligo while T4 and TPOAb had a significant association with age, BMI, and BSA overall. Statistically, T3 was linked with age and BSA overall only. More studies with a higher level of evidence are required to establish the association of Vit-D, zinc, thyroid biomarkers, and thyroid antibodies.

## Introduction

Vitiligo is an autoimmune disease that causes depigmentation of the skin [1]. It can be classified into two major types, segmental (SV) and nonsegmental vitiligo (NSV) [1,2]. Vitamin D (Vit-D) levels have been linked with the development of numerous autoimmune diseases, such as vitiligo, which might contribute to the role of Vit-D in the regulation of both the adaptive and innate immune systems [3,4]. Zinc is also related to the depigmentation process since melanin has a high affinity for metal ions including zinc. Melanosomes are considered storage for metals, and the loss of pigments, theoretically, can increase zinc levels [5]. Thyroid disturbances in biomarkers, i.e., Triiodothyronine (T3), thyroxine (T4), thyroid stimulating hormone (TSH), thyroid peroxidase antibody (TPOAb), thyroglobulin antibody (TGAb), zinc, and Vit-D have been associated with various dermatological manifestations. In addition, Graves’ disease and Hashimoto’s disease have been correlated with vitiligo, as individuals are more prone to thyroid disturbance [6].

The prevalence of vitiligo worldwide has been investigated in a systematic review with a prevalence of 1.8%. Additionally, females have a higher prevalence than males [7]. In a study conducted in Makkah, Saudi Arabia, an incidence of 0.43% was established. Moreover, females were the majority (67.4%), with a female-male ratio of 2.06:1 [8]. Higher Vit-D levels were also correlated with a higher repigmentation rate, and patients are more likely to have a stable form of vitiligo [9]. Zinc is another factor that plays a role in the depigmentation process, which is explained in a study that compared the corticosteroid group versus the zinc sulfate-corticosteroid that manifested responses of 21.43% and 24.7%, respectively [10]. Hashimoto thyroiditis is another autoimmune disease that is associated with vitiligo, which can be attributed to antigen crossover and an increased oxidative stress level [11]. Additionally, TPOAb levels that are associated with Hashimoto disease were significantly higher in vitiligo patients versus the control group (18.1% versus 7.3%, respectively) [12].

Even though previous studies have shown correlations between Vit-D, zinc, TSH levels, and vitiligo, the number of studies that were conducted previously and addressed this topic have substantial limitations, including low levels of evidence, small samples, and insufficient variables [7-12]. This study aims to observe and assess possible associations between numerous variables, such as Vit-D, zinc, and thyroid abnormalities, with vitiligo patients in National Guard Health Affairs (NGHA), Saudi Arabia.

## Materials and methods

A retrospective cohort review of medical records encompassed 297 vitiligo patients who visited dermatology clinics within Saudi Arabian National Guard facilities in the western region of Jeddah, Saudi Arabia, from January 2016 to August 2021. Only patients who had their last hospital visit to the dermatology or family medicine department were included according to their diagnosis of vitiligo through the ICD-10 coding system (L80). Simple random sampling was then used to collect their corresponding file numbers, which were distributed to the data collectors. Data were collected by the coauthors utilizing a unified data collection form. Data collection started in August 2021 and was finalized in December 2021. Patients’ files were assessed for multiple variables that were divided according to their nature, either quantitative or qualitative as follows.

Quantitative variables included age, body mass index (BMI) body surface area (BSA overall) of patients, BSA affected with depigmentation (BSA involved with vitiligo), T3, T4, TSH, zinc, Vit-D, TPOAb, and TGAb. All labs were assessed with the latest available test results. Qualitative variables included sex, and vitiligo types were divided into segmental, nonsegmental, or unclassified, and subtypes were classified as generalized, acral, acrofacial, focal, mucosal, or universal. The latest topical modality of treatment was evaluated with the use of monobenzone, calcineurin inhibitors, corticosteroids, or other modalities of therapy.

Data were extracted from the collection form and entered into a Microsoft Excel spreadsheet for the aggregation of data. Eventually, the data were exported to SPSS V.26.0 (IBM Inc., Armonk, NY) for data analysis.

Descriptive statistics were calculated for all applicable variables and are presented as mean ± standard deviation or percentage, while the median was calculated for variables with extreme outliers. Kruskal-Wallis test, one-way analysis of variance (ANOVA), and independent t-test analysis were utilized for quantitative-qualitative data analysis for all data hypothesizing the data to be normally or abnormally distributed. Chi-square and other variants, i.e., Fisher’s exact test, were used for qualitative-qualitative variable analysis. The correlation of quantitative-quantitative data was evaluated using Pearson’s correlation coefficient. A P-value < 0.05 was considered significant. King Abdullah International Medical Research Centre, National Guard Health Affairs (NGHA), Jeddah, Saudi Arabia granted ethical and research council approval (reference number: JED-21-427780-115144). The research was performed in accordance with The Code of Ethics of the World Medical Association (Declaration of Helsinki).

## Results

The mean age of patients with SV was 29 years, while the NSV group's mean age was 31 years. None of the demographic associations were insignificant. However, NSV in female participants was higher than that in male participants (51.8% versus 36.7%). Additionally, the NSV group showed a higher mean BMI and BSA over SV patients. Most participants were single, unlike those with an unclassified diagnosis, which demonstrates a higher married percentage of participants. Table 1 presents detailed demographic characteristics.

**Table 1:** Demographic characteristics BSA: Body Surface Area, BMI: Body Mass Index *ANOVA test, **Fisher’s exact test No significance was found in this table

			Segmental	Nonsegmental	Unclassified	P-value
Age (years)	Mean ± SD		29 ± 17	31 ± 17	28 ± 18	0.597*
Sex	Male	n (%)	4 (1.3%)	109 (36.7%)	13 (4.37%)	0.630**
	Female	n (%)	4 (1.3%)	154 (51.8%)	13 (4.37%)	
BMI (kg/m^2^)	Mean ± SD		22.54 ± 8.16	25.65 ± 7.26	23.19 ± 7.13	0.148*
BSA (m^2^)	Mean ± SD		1.59 ± 0.43	1.65 ± 0.36	1.51 ± 0.46	0.169*
Social status	Single	n (%)	6 (2.02%)	140 (47.1%)	10 (3.36%)	0.170**
	Married	n (%)	2 (0.6%)	123 (41.4%)	16 (5.3%)	

Significant correlations were demonstrated with a T4 level in association with age, BMI, and overall BSA involved (p>0.001, p=0.006, p=0.003, respectively). Furthermore, TPOAb levels were significantly associated with age (p=0.014), BMI (p>0.001), and overall BSA involved (p=0.001). Likewise, TGAb was significantly correlated with BSA, which is involved in vitiligo (p=0.020). Table 2 demonstrates Vit-D, zinc, and thyroid biomarkers' associations with characteristics of all patients that were included in this research.

**Table 2:** Vit-D, zinc, and thyroid biomarkers associations with the characteristics of age, BMI, and BSA that are involved with vitiligo Vit-D: Vitamin D, TSH: Thyroid-stimulating hormone, T3: Triiodothyronine, T4: Thyroxine, TPOAb: thyroid peroxidase antibody, TGAb: thyroglobulin antibody, C: Pearson’s coefficient, P: P-value (significance) **Correlation is significant at p-value ≤ 0.01, *Correlation is significant at p-value ≤ 0.05

		Age	BMI	BSA overall	BSA Involved with vitiligo
Vit-D (nmol/L)	C	.073	.018	.009	.248
	P	.359	.823	.908	.144
Zinc (mmol/L)	C	.057	.123	.237	-.386
	P	.828	.638	.359	.449
TSH (mIU/L)	C	.010	.044	-.055	.212
	P	.878	.517	.413	.117
T3 (pmol/L)	C	-.490**	-.280	-.364*	.546
	P	.001	.066	.015	.054
T4 (mIU/L)	C	-.348**	-.191**	-.205**	-.186
	P	< .001	.006	.003	.191
TPOAb (IU/ml)	C	.240*	.362**	.312**	.198
	P	.014	< .001	.001	.323
TGAb (IU/ml)	C	.074	-.007	.026	.444*
	P	.458	.945	.795	.020

Vit-D median levels were higher in males (36.8 nmol/L) than in females (33.1 nmol/L). Additionally, lower TSH mean levels were observed in male patients than in female patients (2.86 ± 4.43 mIU/L versus 3.16 ± 7.66 mIU/L). In contrast, zinc mean levels were elevated in male patients more than in females (17.35 ± 8.19 mmol/L versus 9.89 ±2.27 mmol/L). The median T4 in males was higher than that in females (13.22 mIU/L versus 12.79 mIU/L). In contrast to T4, TPOAb was more elevated in female participants (3.02 IU/m) than in male participants (1.78 IU/m). None of these relations is shown to be significant. Table 3 evaluates sex and biomarkers associations.

**Table 3:** The comparison of means of biomarkers among sexes Vit-D: Vitamin D, TSH: Thyroid-stimulating hormone, T3: Triiodothyronine, T4: Thyroxine, TPOAb: Thyroid peroxidase antibody, TGAb: Thyroglobulin antibody *Regarded for mean t: Independent t-test score with equal variance assumed, P: p-value (significance), SD: Standard deviation, KSH: Kruskal Wallis H No significance was found in this table

	Male (n=126)		Female (n=171)		
Biomarker (unit)	Mean ± SD	Median	Mean ± SD	Median	Significance
Vit-D (nmol/L)	43.9 ± 21.7	36.8	38.9 ± 18.2	33.1	t*=1.559 P=0.121 KSH=2.7
Zinc (mmol/L)	17.35 ± 8.19	14.83	9.89 ±2.27	10.27	t*=3.206 P=0.06 KSH=4.063
TSH ( mIU/L)	2.86 ± 4.43	1.90	3.16 ± 7.66	2.12	t*=-0.352 P=0.725 KSH=0.33
T3 (pmol/L)	4.92 ± 0.92	4.80	4.61 ± 0.93	4.60	t*=1.084 P=0.285 KSH=1.017
T4 (mIU/L)	13.32 ±2.18	13.22	13.01±1.89	12.79	t*=1.103 P=0.271 KSH=0.521
TPOAb (IU/ml)	146.27±371.69	1.78	105.62 ± 298.21	3.02	t*=0.621 P=0.536 KSH=0.057
TGAb (IU/ml)	134.30 ± 445.61	3.81	97.84 ± 293.20	97.84	t*=0.504 P=0.616 KSH=1.415

Vit-D mean levels were the highest in the universal subtype (52.2 ± 22.3 nmol/L), followed by the acral and focal subtypes (43.6 ± 28.0 nmol/L, 43.6 ± 18.2 nmol/L), while the lowest level was observed in the mucosal subtype (31.3 ± 6.6 nmol/L). Additionally, TSH mean levels were also highest in universal vitiligo (7.21 ± 19.53 mIU/L) and lowest in acrofacial vitiligo (1.96 ± 1.30 mIU/L). The mean levels of TPOAb showed acral vitiligo to be the highest (311.59 ± 719.78 IU/m), followed by unclassified vitiligo (155.08 ± 224.08 IU/m). Additionally, TPOAb mean levels in mucosal vitiligo were the lowest (2.62 ± 2.67 IU/m). NSV subtypes show an increased mean level of TGAb compared with SV specifically universal, as it shows the highest mean level (158.77 ± 332.78 IU/mL). Additionally, the highest zinc mean levels were demonstrated in acrofacial vitiligo (17.73 ± 12.40 mmol/L). The remaining biomarkers are shown in the table of which none are shown to be significant. Table 4 illustrates the types of vitiligo and associated patient biomarkers.

**Table 4:** The types of vitiligo and associated patient biomarkers Vit-D: Vitamin D, TSH: Thyroid-stimulating hormone, T3: Triiodothyronine, T4: Thyroxine, TPOAb: Thyroid peroxidase antibody, TGAb: Thyroglobulin antibody, P: p-value All values are represented as mean ± standard deviation (Mean rank of Kruskal Wallis) ANOVA test was used for this table *No patients were found with matched data, **Sample is insufficient to calculate standard deviation No significance was found in this table

	Segmental	Non-Segmental						Unclassified	P-value of comparison of means between vitiligo types
		Generalized	Acral	Acrofacial	Focal	Mucosal	Universal		
Vit-D (nmol/L)	32.5 ± 8.9 (64.40)	39.4 ± 18.7 (66.21)	43.6 ± 28.0 (69.21)	38.6 ±13.6 (69.73)	43.6 ± 18.2 (78.96)	31.3 ± 6.6 (56.75)	52.2 ± 22.3 (95.03)	35.6 ± 10.1 (74.38)	P=0.375
		Comparison of means between vitiligo subtypes P=0.313							
Zinc (mmol/L)	N/A*	10.08 ± 2.45 (8.36)	N/A*	17.73 ± 12.40 (10.50)	7.77** (3.00)	10.62** (9.00)	10.89**(11.00)	14.83** (16.00)	P=0.434
		Comparison of means between vitiligo subtypes P=0.291							
TSH (mIU/L)	2.21 ± 1.43 (102.14)	2.40 ± 1.74 (100.69)	2.68 ± 2.74 (98.59)	1.96 ± 1.30 (84.39)	5.38 ± 9.95 (108.12)	2.59 ± 0.58 (125.10)	7.21 ± 19.53 (122.03)	2.70 ± 1.77 (124.83)	P=0.921
		Comparison of means between vitiligo subtypes P=0.57							
T3 (pmol/L)	N/A*	4.78 ± 0.79 (23.04)	4.51 ± 1.30 (19.57)	4.41 ± 1.08 (19.33)	4.28 ± 0.61 (14.70)	N/A*	5.30 ± 1.00 (29.50)	6.10** (41.00)	P=0.140
		Comparison of means between vitiligo subtypes P=0.422							
T4 (mIU/L)	13.25 ± 0.37 (116.30)	13.13 ± 1.90 (96.35)	13.31 ± 1.61 (107.00)	12.38 ± 1.47 (74.12)	12.83 ± 1.57 (87.41)	14.32 ± 1.81 (132.30)	12.84 ± 3.74 (79.71)	14.20 ± 1.92 (137.38)	P=0.146
		Comparison of means between vitiligo subtypes P=0.451							
TPOAb (IU/ml)	9.07 ± 13.97 (40.33)	110.72 ± 266.46 (50.80)	311.59 ± 719.78 (41.50)	43.82 ± 94.82 (46.73)	121.53 ± 289.79 (48.67)	2.62 ± 2.67 (37.00)	121.36 ± 282.20 (46.00)	155.08 ± 224.08 (62.57)	P=0.812
		Comparison of means between vitiligo subtypes P=0.433							
TGAb (IU/ml)	2.54 ± 2.14 (22.50)	146.80 ± 477.25 (46.45)	79.68 ± 83.58 (57.80)	54.39 ± 115.27 (49.50)	33.94 ± 66.68 (42.17)	14.84 ± 13.99 (45.25)	158.77 ± 332.78 (42.57)	140.76 ± 257.91 (64.71)	P=0.853
		Comparison of means between vitiligo subtypes P=0.917							

**Figure 1:**
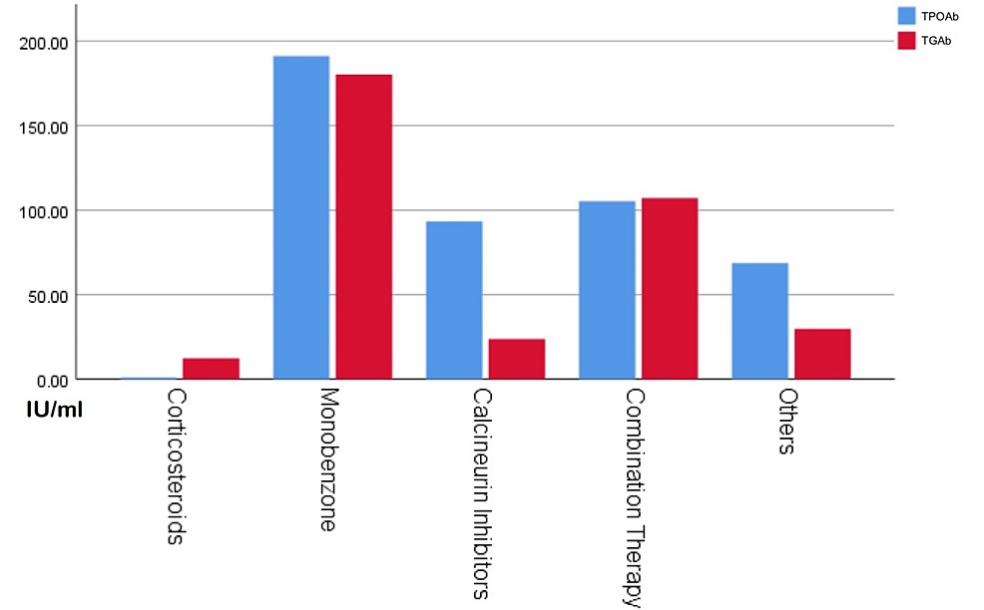
Thyroid antibodies' levels in different topical modalities of treatment TPOAb: thyroid peroxidase antibody, TGAb: thyroglobulin antibody No significance was found in this figure

Eight patients were using corticosteroids while 37 were prescribed calcineurin inhibitors as monotherapy. One hundred fifty-eight used a combination of both. Sixty-nine patients were prescribed monobenzone depigmentation therapy of 25 patients were treated with other modalities of treatment. No association was found in means of TGAb and TPOAb among different modalities of treatment. Monobenzone as a modality of treatment is associated with the highest mean levels of both TGAb and TPOAb, followed by combination therapy. Moreover, calcineurin inhibitors are the third in terms of mean levels of TPOAb. Additionally, corticosteroids demonstrate the lowest mean levels of both TGAb and TPOAb. Though the comparison was insignificant, it is sufficiently high to raise questions regarding its association. Figure 1 compares the latest topical treatment modality and thyroid biomarkers' levels.

## Discussion

Vitiligo is an acquired pigmentary disorder characterized by circumscribed depigmented areas of skin and mucous membranes. It frequently appears in childhood or early adulthood. This disease has an enormous cosmetic impact along with a significant psychological impact [13,14]. In addition, a variegated form of vitiligo is classified into two types: SV and NSV. NSV is classified according to the pattern of involvement: focal, mucosal, generalized, acrofacial, acral, and universal [14-16]. The study aims to observe the association of numerous factors, such as Vit-D, zinc, and thyroid abnormalities, with vitiligo patients in the National Guard Health Affairs (NGHA) in Jeddah, Saudi Arabia.

The results showed that the sample with NSV have a higher BMI than those with SV (25.65 ± 7.26 compared to 22.54 ± 8.16). Moreover, the analysis confirmed that the SV group had a lower BSA than NSV participants (1.59 ± 0.43 in comparison with 1.65 ± 0.36). In addition, the data suggest that the NSV cohort tends to be older than SV patients (31 ± 17 compared to 29 ± 17). In terms of sex, the study demonstrated that the percentage of NSV in females was elevated to that in males (51.8% versus 36.7%). Very low correlations were noticed between TSH level and age, BMI, and BSA overall, and no significant associations were established.

Moreover, Moghaddam et al reported lower zinc levels in subjects with vitiligo [13]. Mirnezami and Rahimi also reported low serum zinc levels in generalized patients with vitiligo [14]. Our chart review demonstrated a nonsignificant correlation between zinc level and BSA involved with vitiligo with a Pearson’s coefficient of -0.386. Additionally, T4 demonstrated significant correlations with age, BMI, and BSA involved (p>0.001, p=0.006, p=0.003, respectively).

The results from our research showed that Vit-D levels were lower in females with a mean of 36.8 nmol/L versus males with a mean of 33.1 nmol/L, comparably, a study conducted in Iran presented increased Vit-D levels in females compared to males with a mean of 26.16 ± 17.11 ng/mL versus 22.37 ± 10.78 ng/mL [16]. In addition, previous research conducted on a total of 150 vitiligo patients in Riyadh, Saudi Arabia, demonstrated that serum levels of Vit-D among vitiligo patients were lower in males (p=0.01) [3]. Similarly, higher TSH mean levels were observed in females compared to males in both our article (2.86± 4.43 versus 3.16 ± 7.66 mIU/L) and the study conducted in Iran (4.01 ± 0.57 mIU/L versus 3.89 ± 0.74 mIU/L). In addition, zinc mean levels were excessive in males compared to females, respectively (17.35 ± 8.19 mmol/L, 9.89 ± 2.27 mmol/L); likewise, zinc means ± SD in the probe conducted in Sina Hospital, Tabriz, Iran, was elevated in males with a mean ± SD of 90.94 ± 20.88 compared to females with 84.68 ± 21.08 [16]. The Differences between levels of Vit-D among males and females in our paper in relation to other studies could be due to demographical factors, lifestyle, and economical variations involved in this research in contrast to other related studies. For example, this retrospective cohort paper included 297 patients with vitiligo, 58% of whom were females and 42% of whom were males. In contrast, the research conducted in Tabriz, Iran, involved more male patients, with 98 patients with vitiligo showing a percentage of 51% male and 49% female. Moreover, a published article conducted in Riyadh, Saudi Arabia, involving 150 vitiligo patients reflected a percentage of up to 60% of males [3,16].

This article is subject to a small number of limitations. One of which is the fact that it was based on data from a single hospital center in Jeddah, Saudi Arabia, and could not include all participants with vitiligo in the general population. The main disadvantage is that our report was restricted to subjects of National Guard Health Affairs and may not represent all vitiligo patients who are treated in other public hospitals in Saudi Arabia, which shows a lack of diversity regarding ethnicity and socioeconomic status among the included patients. Another drawback is that only the latest available laboratory results were used, and some of these tests were not recently ordered. The absence of a control group is also a large pitfall. Lastly, T3 and zinc levels were not as available in other laboratory tests, which made it difficult to calculate their corresponding means and standard deviations; thus, establishing conclusions was not applicable. Despite these weaknesses, the strengths of our paper outweigh its limitations. This is the first study performed in Saudi Arabia regarding the association between Vit-D, zinc, and thyroid abnormalities with vitiligo. As a result of our findings, we advise frequent screening of thyroid function, anti-thyroid antibodies, and Vit-D levels for patients with vitiligo as recommended by the British Association of Dermatologists guidelines for the management of vitiligo. For future research, we recommend considering prospective studies with larger sample sizes involving the public to obtain optimal findings regarding this topic. In addition, we recommend involving a control group, patients with recent laboratory tests, and including more patients with available T3 and zinc tests to inspect their association with vitiligo.

## Conclusions

This article concludes that vitiligo has no affiliation with regard to demographic variables. However, a connection between age, thyroid biomarkers, and antibodies was found. Age and BMI are associated with T4 and TPOAb. Vit-D and Zinc did not have a linkage to vitiligo. Vitiligo involving extensive BSA is related to TGAb. Patients using monobenzone recorded high nonsignificant TPOAb and TGAb levels. Since the majority of these correlations were highly significant, we conclude that thyroid biomarkers and antibodies should be measured and observed during the course of vitiligo treatment and follow-up. Higher evidence-controlled studies are needed to support our findings.
